# Practical Departments

**Published:** 1897-09-11

**Authors:** 


### PRACTICAL DEPARTMENTS.

### A PORTABLE SHOWER BATH.

One of the most useful inventions we have seen for some time is a portable shower bath invented by Mr. W. H. Slade, 17, Duke Street, Bristol. The arrangement consists of a circular tube, perforated with small holes on the lower side, and having attached to it a length of tubing with a patent nozzle which fits on to any ordinary tap. When the tubing has been attached to the tap the user puts the ring over his head with the perforated side downwards, turns on the tap, and immediately the shower flows over his body. With this ring shower bath the head is not wet, which will probably be a recommendation in the eyes of ladies, who find not only the ordinary shower but even the spray bath ruinous to the *coiffure*. As the whole arrangement, tube, ring, and nozzle complete, does not weigh quite two pounds, it can be included in anybody's luggage, and then, given a water tap and a tub of any kind, a shower bath is at one's command. The portable shower bath ring is made in three styles. The first costs 7s. 6d., and consists of a lacquered ring with the tubing attached. The next style, at 10s. 6d., has the ring nickel-plated, and the tube is detachable, giving greater convenience in packing. The third style adds to this advantage a further one. In it the tubing is forked at the further end; the forks can be attached to the hot and cold taps of the ordinary bath simultaneously, and a tepid shower enjoyed at any temperature the user likes, according to the extent to which he turns on either tap. This invention seems to us admirably convenient. To tourists it would be especially valuable, and we think nurses would find it useful both for themselves and for patients. In the poorest house there is now a cold water tap; in those of very modest pretensions there is hot and cold water. Given a water supply, a tub, and one of these rings, and a refreshing shower-bath is at any one's command.

